# Supplementary material for: Positive association between Brucella spp. seroprevalences in livestock and humans from a cross-sectional study in Garissa and Tana River Counties, Kenya
Source: PLoS Negl Trop Dis. 2019 Oct 17;13(10):e0007506. doi: 10.1371/journal.pntd.0007506 (PMC6818805; doi:10.1371/journal.pntd.0007506)
Supplement: S1 Checklist — (DOC) [file pntd.0007506.s001.doc]

STROBE Statement—Checklist of items that should be included in reports of ***cross-sectional studies***

|  | Item No | Recommendation |
| --- | --- | --- |
| **Title and abstract** | 1 | (*a*) Cross sectional study |
|  |  | (*b*) Abstract 18 - 48 |
| Introduction | | |
| Background/rationale | 2 | Background: Introduction, paragraph 1 – 2, lines 66 - 87  Rationale: Introduction, paragraph 3, lines 89 - 102 |
| Objectives | 3 | Introduction, paragraph 4, lines 104 - 108 |
| Methods | | |
| Study design | 4 | Methods, paragraph 2-3, lines 124 - 148 |
| Setting | 5 | The setting and location: methods paragraph 1, lines 112 - 121 |
| Participants | 6 | (*a*) Eligibility criteria: Sources and methods for selecting participants: methods, lines 144- 146 |
| Variables | 7 | Methods, lines 219 - 231 |
| Data sources/ measurement | 8* | Methods, lines 151 - 209 |
| Bias | 9 | Multivariable analyses, adjustment for clustering, lines 235 -242 |
| Study size | 10 | Methods, lines 141 - 142 |
| Quantitative variables | 11 | Age, lines 225 – 227 |
| Statistical methods | 12 | (*a*) Methods, lines 233 - 242 |
|  |  | (*b*) NA |
|  |  | (*c*) NA |
|  |  | (*d*) NA |
|  |  | (*e*) NA |
| Results | | |
| Participants | 13* | (a) Results, paragraph 1 lines 272-273 and 285 - 286 |
|  |  | (b) NA |
|  |  | (c) NA |
| Descriptive data | 14* | (a) Results, Table 1 and Table 2 |
|  |  | (b) NA |
| Outcome data | 15* | Results, 297 – 313 |
| Main results | 16 | (*a*) Tables 3 - 5 |
|  |  | (*b*) NA |
|  |  | (*c*) NA |
| Other analyses | 17 | NA |
| Discussion | | |
| Key results | 18 | Discussion, lines 392 - 401 |
| Limitations | 19 | Discussion, lines 464 - 473 |
| Interpretation | 20 | Discussion, lines 403 - 424 |
| Generalisability | 21 | Discussion, lines 447 – 462 |
| Other information | | |
| Funding | 22 | Funding – submission form |

*Give information separately for exposed and unexposed groups.

**Note:** An Explanation and Elaboration article discusses each checklist item and gives methodological background and published examples of transparent reporting. The STROBE checklist is best used in conjunction with this article (freely available on the Web sites of PLoS Medicine at http://www.plosmedicine.org/, Annals of Internal Medicine at http://www.annals.org/, and Epidemiology at http://www.epidem.com/). Information on the STROBE Initiative is available at www.strobe-statement.org.
